# Author Correction: Geometric Control of Cell Migration

**DOI:** 10.1038/s41598-018-33004-x

**Published:** 2018-10-16

**Authors:** Bo Chen, Girish Kumar, Carlos C. Co, Chia-Chi Ho

**Affiliations:** 1 Chemical & Materials Engineering Program, University of Cincinnati, Cincinnati, OH 45221-0012 USA; 2 Division of Biology, Center for Devices and Radiological Health, US Food & Drug Administration, Silver Spring, MD 20993 USA

Correction to: *Scientific Reports* 10.1038/srep02827, published online 3 October 2013

The following information was omitted from the Methods section.

The images in Figures 2, 3B, and 4A were captured through a coverslip. To achieve this, we cut the boundary of tissue culture dishes and mounted with fluoromount-G. The contrast/brightness of the Golgi stain in Figure 5 was adjusted using Image J software which can alter certain details of the Golgi structure. However, the key point was to show the brief position of the Golgi apparatus during the release period. They are not relative to the certain details of the Golgi structure.

